# Four-year antibody persistence and response to a booster dose of a pentavalent MenABCWY vaccine administered to healthy adolescents and young adults

**DOI:** 10.1080/21645515.2018.1457595

**Published:** 2018-05-09

**Authors:** Xavier Sáez-Llorens, Johnny Beltran-Rodriguez, Jose M. Novoa Pizarro, Ilhem Mensi, Pavitra Keshavan, Daniela Toneatto

**Affiliations:** a Hospital del Niño “Dr. José Renán Esquivel”, Infectious Disease Department, Panama City, Panama and distinguished investigator of the SNI (Senacyt, Panama); b Centro de Atención e Investigación Médica Caimed. Bogotá, Colombia; c Faculty of Medicine University of Desarrollo/Clinica Alemana, Santiago, Chile; d GSK, Amsterdam, The Netherlands; e GSK, Siena, Italy

**Keywords:** antibody persistence, booster dose, MenACWY, MenABCW, meningococcal vaccine

## Abstract

This open-label, multicenter extension study (NCT02451514) assessed persistence of *Neisseria meningitidis* serogroups ABCWY antibodies 4 years after primary vaccination. Adolescents and young adults who previously received 2 doses of MenABCWY+OMV (Group III), 1 dose of MenACWY-CRM (Group VI), or newly-recruited vaccine-naïve participants (Group VII) were administered 1 (Group III) or 2 doses (Groups VI and VII) of MenABCWY+OMV, 1 month apart. Immunogenicity was assessed by human serum bactericidal assay (hSBA). Safety and reactogenicity were also evaluated. Percentages of participants with hSBA titers ≥8 (serogroups ACWY), ≥5 (serogroup B) and hSBA geometric mean titers (GMTs) were evaluated in all 129 enrolled participants (Group III: 33; Group VI: 46; Group VII: 50). Anti-ACWY antibody concentrations waned over 4 years post-vaccination, but remained above pre-vaccination concentrations. Similarly, levels of antibodies against serogroup B test strains also waned over 4 years post-vaccination, but remained above pre-vaccination concentrations for some strains. MenABCWY+OMV booster induced a robust anamnestic anti-ACWY response in Group III and VI and a good response against serogroup B test strains (≥82%) in Group III. In serogroup B-naïve participants (Groups VI and VII), anti-B responses to 2 doses of MenABCWY+OMV were less homogenous and lower than in Group III. MenABCWY+OMV was reactogenic, but well-tolerated. No safety concerns were identified. These findings indicate that although antibodies against *N. meningitidis* serogroups ABCWY waned over 4 years post-vaccination, exposure to a MenABCWY+OMV booster dose elicits an anamnestic response in adolescents previously exposed to the same or another multivalent meningococcal vaccine.

## Introduction

Invasive meningococcal disease (IMD) caused by *Neisseria meningitidis* is the leading cause of bacterial meningitis and sepsis in the US and Europe.^1^^,^^2^ Due to the fulminant nature of the disease, IMD has high morbidity and mortality rates in children and adolescents, even when patients receive early antibiotic treatment.^1^ IMD survivors have an 11%–19% risk of suffering physical, cognitive or neurological sequelae.^3^ IMD incidence is highest in infants, but a second smaller peak occurs in adolescents and young adults due to behavioral and lifestyle-associated risk factors.^4^^,^^5^

IMD incidence varies globally. This variation is largely driven by the distribution of *N. meningitidis* serogroups A, B, C, W, X and Y, which are responsible for most of the meningococcal disease cases worldwide.^1^^,^^6^ In Latin America, for example, serogroups B and C are responsible for the majority of IMD cases. Additionally, an emergence of serogroups W and Y was recently reported in Argentina, Brazil, Chile, Paraguay, Uruguay, Colombia and Venezuela.^7-10^ Although IMD incidence is difficult to assess in Latin America due to poor surveillance systems in some regions, current estimates of disease incidence are <0.1 to 2 cases per 100,000.^11^

To reduce the burden of IMD, monovalent and multivalent vaccines against serogroups A, C, W, and Y have been included in vaccination programs since 1999.^12^^,^^13^ In Latin America, meningococcal vaccines are included in national immunization programs only in Argentina, Cuba, Brazil and Chile.^5^^,^^14-16^ Meningococcal quadrivalent MenACWY vaccines, such as MenACWY-CRM (*Menveo*, GSK) are approved in Argentina, Brazil, Chile and Colombia for adolescent vaccination.^15-18^

Multi-component recombinant meningococcal vaccines have been developed to target serogroup B. 4CMenB (*Bexsero*, GSK) is currently approved in more than 35 countries worldwide, including Chile, Brazil and Uruguay.^19^ 4CMenB combines 3 recombinant proteins (factor H binding protein [fHbp], *Neisseria* adhesin A [NadA] and Neisserial heparin binding antigen [NHBA]) with outer membrane vesicles (OMV) from the New Zealand outbreak strain NZ98/254 (porin A, [PorA]).^20^^,^^21^ In phase 2 and phase 3 clinical studies, 4CMenB was well tolerated and elicited strong bactericidal responses,^22-24^ also when co-administered with routine childhood vaccines^25^ or with quadrivalent meningococcal ACWY conjugate vaccine to laboratory workers.^26^^,^^27^

Development of a combined vaccine against *N. meningitidis* serogroups A, C, W, Y and B would simplify the vaccination schedule for the 5 serogroups and possibly increase compliance. A phase 2 randomized controlled study conducted in Panama, Colombia, and Chile evaluated safety and immunogenicity of 4 different MenABCWY vaccine candidates in healthy adolescents.^28^ The candidate vaccine that was selected for further investigation is composed of MenACWY conjugated to a carrier protein cross reactive material 197 (CRM_197_) combined with recombinant proteins from serogroup B and outer membrane vesicle from New Zealand strain NZ98/254 (MenABCWY+OMV). In the primary study,^28^ 2 doses of the investigational MenABCWY+OMV vaccine given 2 months apart induced substantial immune responses against all serogroups and the candidate vaccine was well-tolerated.^28^ In a first follow-up extension study,^29^ the immune responses against serogroups A, C, W and Y persisted for up to 12 months. However, levels of antibodies against serogroup B test strains waned by 6 months after vaccination and then stabilized up to 12 months.^29^ A sub-population receiving a booster third dose of MenABCWY+OMV 6 months after vaccination showed increased immune responses against all serogroups evaluated at 6 months after the booster dose.^29^

Studies conducted in adolescents and adults show that the immune response to a single dose of MenACWY-CRM persists up to 5 years after vaccination and that a booster dose of MenACWY-CRM 3-5 years after primary vaccination elicits a strong immune response in nearly all vaccinees.^30-32^ Similarly, the immune response to the 4CMenB vaccine also persists to a varying degree across strains, for up to 4 years and a booster dose elicits an immune response when administered 4 years post-primary vaccination.^33^ Currently, no data are available on long-term antibody persistence and booster response for the MenABCWY+OMV vaccine. These data would be valuable for assessing the duration of the immune response, the need for a booster and vaccine suitability in outbreak management.

In this second extension study, we assessed 4-year persistence after 2 doses of MenABCWY+OMV and the response to a booster dose against *N. meningitidis* serogroups A, B, C, W and Y. We also evaluated the antibody response after 2 doses of MenABCWY+OMV in adolescents and young adults previously vaccinated with a single dose of the MenACWY-CRM vaccine and in vaccine-naïve participants of matching age.

**Figure 1.**
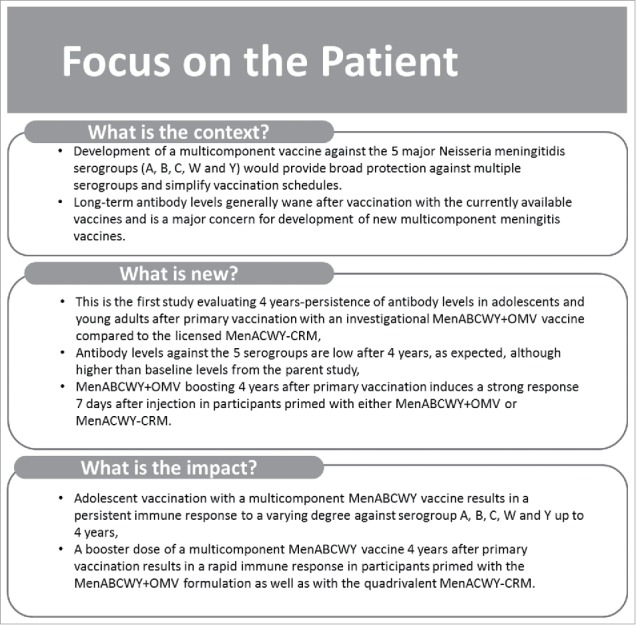
Focus on the patient section.

A summary contextualizing the results and potential clinical research relevance and impact is displayed in the Focus on the Patient Section (Fig. 1).

## Results

### Demographics

**Figure 2.**
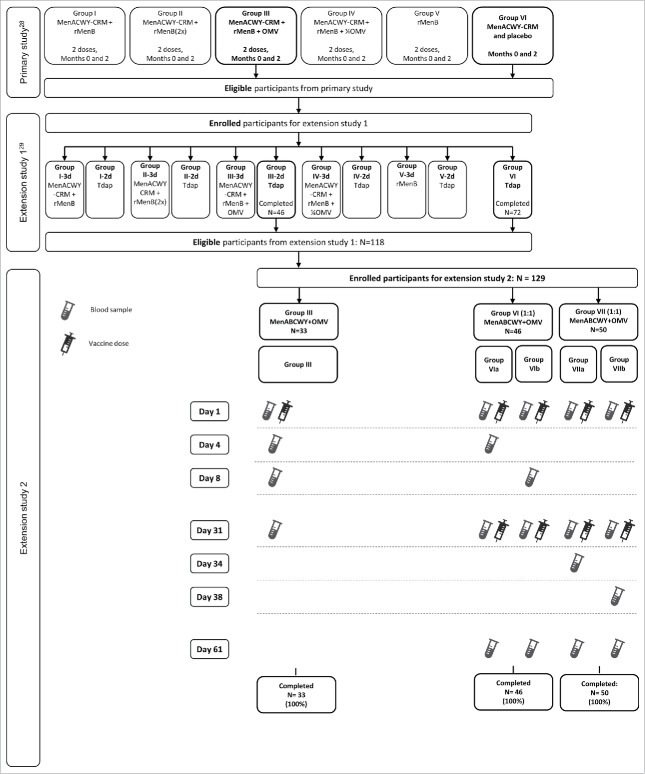
Flow of participants Footnote: Primary study: participants received 1 or 2 doses of vaccine; Extension study 1: Follow-on participants for 10 months persistence, 3^rd^ dose administration (with control vaccination); Extension study 2: Follow-on participants for 4 years persistence, 3rd dose administration (with control vaccination). Group III; participants received 2 doses of MenABCWY+OMV (presented in this figure, for primary and extension 1 studies, as MenACWY-CRM + rMenB+OMV) in the primary study and 1 dose of MenABCWY+OMV in the extension study presented here (4 years post-primary study). Group VI; participants received 1 dose of MenACWY-CRM in the primary study and 2 doses, 1 month apart, of MenABCWY+OMV in the extension study presented here (4 years post-primary study). Group VII; participants were newly recruited and naïve to vaccination against A, B, C, W and Y serogroups of *N. meningitidis*. They were vaccinated with 2 doses, 1 month apart, of MenABCWY+OMV in the extension study presented here.

Out of the 485 eligible participants who completed the primary study,^28^ 79 participants were enrolled in this second extension, with 33 participants who previously received 2 doses of MenABCWY+OMV (Group III), and 46 participants who received 1 dose of MenACWY-CRM (Group VI). This extension also included 50 newly-recruited non-vaccinated participants (Group VII). All 129 participants completed the extension study (Fig. 2). Demographic and other baseline characteristics were similar in all groups (Table 1).

**Table 1.** Study population demographics and baseline characteristics (FAS Persistence).

	Group III	Group VI	Group VII	Total
	N=33	N=46	N=50	N=129
Age, years (Mean ±SD)	18.6 ± 2.21	18.7 ± 1.87	18 ± 2.04	18.4 ± 2.04
Sex, n (%)				
Male	11 (33%)	19 (41%)	25 (50%)	55 (43%)
Female	22 (67%)	27 (59%)	25 (50%)	74 (57%)
Ethnicity, n (%)				
Hispanic or Latino	32 (97%)	46 (100%)	50 (100%)	128 (99%)
Not Hispanic or Latino	1 (3%)	0	0	1 (1%)
Weight, kg (Mean ± SD)	68.17 ± 23.136	65.71 ± 16.372	64.97 ± 14.185	66.05 ± 17.514
Height, cm (Mean ± SD)	163.8 ± 8.27	163.1 ± 8.46	166.3 ± 10.01	164.5 ± 9.1
BMI, kg/m^2^ (Mean ± SD)	25.29 ± 7.7	24.51 ± 4.744	23.48 ± 4.724	24.31 ± 5.64

Footnote: FAS, full analysis set; N, number of participants; SD, standard deviation; n (%), number (percentage) of participants in a certain category; BMI, body mass index.

### Immunogenicity

#### Antibody persistence

**Figure 3.**
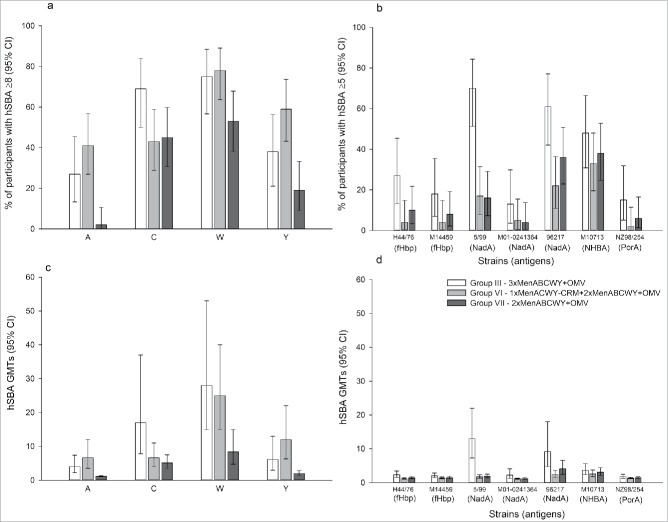
Percentages of participants with hSBA ≥8 against serogroups A, C, W, and Y (a) and hSBA ≥5 against serogroup B (c) and hSBA geometric mean titers against serogroups A, C, W and Y (b) and serogroup B (d) at day 1 (FAS Persistence) Footnote: FAS, full analysis set; hSBA, human serum bactericidal assay, GMT, geometric mean titer; CI, confidence interval. Group III; participants received 2 doses of MenABCWY+OMV in the primary study and 1 dose of MenABCWY+OMV in the extension study presented here (4 years post-primary study). Group VI; participants received 1 dose of MenACWY-CRM in the primary study and 2 doses, 1 month apart, of MenABCWY+OMV in the extension study presented here (4 years post-primary study). Group VII; participants were newly recruited and naïve to vaccination against A, B, C, W and Y serogroups of *N. meningitidis*. They were vaccinated with 2 doses, 1 month apart, of MenABCWY+OMV in the extension study presented here. The group description in the legend includes all doses administered in the primary and extension studies.

Across serogroups, antibody level decreased at 4 years after primary vaccination but still remained higher than the baseline of primary study. The percentages of participants with human serum bactericidal assay (hSBA) titers ≥8 against serogroups A, C, W and Y, 4 years post-primary vaccination, were higher in groups primed with MenACWY-containing vaccines (Group III and Group VI) compared to the vaccine-naïve participants in Group VII (Fig. 3a). Compared to participants primed with 2 doses of MenABCWY+OMV (Group III), more participants primed with 1 dose of MenACWY-CRM (Group VI) showed hSBA titers ≥8 for serogroups A (41% vs 27%) and Y (59% vs 38%), whereas more participants in Group III had a titer ≥8 for serogroup C compared to participants in Group VI (69% vs 43%). Response rates were similar between groups for serogroup W (75% and 78%; Fig. 3a).

hSBA geometric mean titers (GMTs) were also higher in ACWY-primed participants in Groups III and VI, compared to the vaccine-naïve participants in Group VII, with the exception of serogroup C GMTs in Group VI (Fig. 3c). Compared to participants in Group VI, participants in Group III showed numerically higher hSBA GMTs for serogroup C (geometric mean ratio [GMR]: 2.58) and lower hSBA GMTs for serogroups A (GMR: 0.61) and Y (GMR: 0.52). GMTs for serogroup W were similar (GMR: 1.14; Fig. 3c).

Geometric mean concentrations of antibodies against A, C, W and Y serogroups, measured by enzyme-linked immunosorbent assay (ELISA), in Groups III and VI, markedly increased after primary vaccination and declined over the course of 4 years, with antibody concentration remaining higher than the baseline concentrations measured before vaccination in the primary study (Table S1).

Antibody titers against serogroup B test strains waned by 4 years after primary vaccination. The percentages of participants with hSBA titers ≥5 were low (≤48%) in all groups, except for the NHBA test strain (48%) and 2 NadA test strains (5/99 [70%] and 96217 [61%]) in primed participants (Group III). Although 95% confidence intervals (CIs) overlap, percentages of participants with hSBA titers ≥5 were consistently higher in Group III compared to non-primed participants in Groups VI and VII for all serogroup B strains (Fig. 3b).

hSBA GMTs 4-years after primary vaccination were low for all serogroup B test strains in both Group III and Group VI, with the exception of the NadA test strain 5/99 (Fig. 3d).

#### MenABCWY+OMV post-booster/dose 1 response

**Figure 4.**
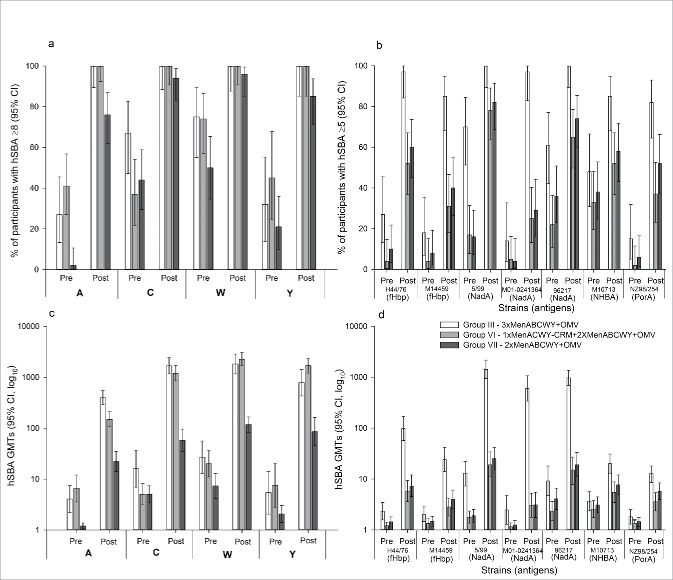
Percentages of participants with hSBA ≥8 against serogroups A, C, W, and Y (a) and hSBA ≥5 against serogroup B (c) and hSBA geometric mean titers by serogroups A, C, W and Y (b) and serogroup B (d) pre-vaccination and at day 31 post-vaccination (FAS Day 31) Footnote: FAS, full analysis set; hSBA, human serum bactericidal assay; GMT, geometric mean titer; CI, confidence interval. Group III; participants received 2 doses of MenABCWY+OMV in the primary study and 1 dose of MenABCWY+OMV in the extension study presented here (4 years post-primary study). Group VI; participants received 1 dose of MenACWY-CRM in the primary study and 2 doses, 1 month apart, of MenABCWY+OMV in the extension study presented here (4 years post-primary study). Group VII; participants were newly recruited and naïve to vaccination against A, B, C, W and Y serogroups of *N. meningitidis*. They were vaccinated with 2 doses, 1 month apart, of MenABCWY+OMV in the extension study presented here. The figure presents results of pre- and post-booster/first dose. The group description in the legend includes all doses administered in the primary and extension studies.

All participants primed with MenACWY antigens (Groups III and VI) achieved hSBA titers ≥8 against serogroups A, C, W and Y at 1 month after vaccination with 1 dose of MenABCWY+OMV. At 1 month post-dose 1 with MenABCWY+OMV, most vaccine-naïve (76%–96%) participants in Group VII achieved hSBA titers ≥8 (Fig. 4a).

At 1 month post-booster/dose 1, hSBA GMTs against serogroups A, C, W and Y increased compared to pre-vaccination GMTs. The relative increase was highest for primed participants in Groups III (GMR range: 67–162) and VI (GMR range: 22–238) compared to vaccine-naïve participants in Group VII (GMR range: 12–46). GMTs were also higher in participants in Groups III and VI, compared to Group VII (Fig. 4c).

After a booster dose of MenABCWY+OMV, hSBA GMTs in participants in Group III, who received 2 doses of MenABCWY+OMV, were higher for serogroups A and C, and lower for serogroups W and Y, compared to participants in Group VI who received 1 dose of MenACWY-CRM (Fig. 4c).

One month post-booster/dose 1, the percentage of participants with hSBA titers ≥5 against serogroup B test strains increased in all groups compared to pre-vaccination percentages, with a higher increase in participants primed against serogroup B (Group III; Fig. 4b).

At 1 month post-booster/dose 1, hSBA GMTs increased over pre-vaccination GMTs in participants primed with MenABCWY+OMV (Group III), in particular against the fHbp test strain H44/76 (GMR: 42), and in all the NadA test strains (5/99 [GMR: 112], M01-0240364 [GMR: 229] and 96217 [GMR: 106]) (Fig. 4d). The increase of hSBA GMTs was more modest in participants not primed with serogroup B test strains (Groups VI and VII), with the highest GMT increases against NadA test strains 5/99 (GMR: 11 and 13 for Group VI and Group VII, respectively) and 96217 (GMR: 6 and 5 for Group VI and Group VII, respectively) (Fig. 4d). For all serogroup B test strains, hSBA GMT values at 1 month post-booster/dose 1 were higher in the participants in Group III, who had received the MenABCWY+OMV vaccine during the primary study, compared to participants in Groups VI and VII, who were naïve for serogroup B vaccination.

### Immunogenicity kinetics after 1 and 2 doses of MenABCWY+OMV

Kinetics of the immune response against serogroups A, C, W and Y and against serogroup B test strains after 1 (Group III and Group VI) and 2 (Group VII) doses of MenABCWY+OMV were determined (Tables 2 and 3).

**Table 2.** Percentages of participants with hSBA ≥ 8 and geometric mean hSBA titers (95% CI) against serogroups ACWY at different timepoints – FAS Immunogenicity Kinetics.

			% of participants with hSBA≥8 (95% CI)					GMT (95% CI)		
			Group III	Group VI		Group VII		Group III	Group VI	Group VII
				a	b	a	b			
Serogroup	Timepoint			N=33	N=23	N=23	N=26	N=24	N=33	N=46	N=50
A	Booster/Dose 1	Pre	27 (13.3–45.5)	52 (30.6–73.2)	30 (13.2–52.9)	0 (0–13.2)	4 (0.11–21.1)	4.06 (2.22–7.44)	6.64 (3.56–12)	1.20 (1.05–1.37)
		D3	24 (11.1–42.3)	52 (30.6–73.2)	—	—	—	3.85 (2.00–7.42)	9.69 (3.90–24)^*^	—
		D7	100 (89.4–100)	—	82 (59.7–94.8)	—	—	474 (321–701)	61 (25–146)^*^	—
		D30	100 (89.4–100)	100 (85.2–100)	100 (85.2–100)	73 (52.2–88.4)	79 (57.8–92.9)	396 (288–545)	149 (106–210)	22 (14–35)
	Dose 2	D3	—	—	—	77 (56.4–91.0)	—	—	—	31 (15–67)^**^
		D7	—	—	—	—	96 (78.9–99.89)	—	—	107 (64–179)^***^
		D30	—	100 (85.2–100)	100 (85.2–100)	100 (85.2–100)	88 (67.6–97.3)	—	33 (20–56)	74 (51–108)
C	Booster/Dose 1	Pre	69 (50.0–83.9)	61 (38.5–80.3)	26 (10.2–48.4)	40 (21.1–61.3)	50 (29.1–70.9)	17 (7.80–37)	6.62 (4.10–11)	5.11 (3.49–7.48)
		D3	73 (54.1–87.7)	64 (40.7–82.8)	—	—	—	21 (9.93–43)	12 (5.13–29)^*^	—
		D7	100 (88.1–100)	—	100 (82.4–100)	—	—	2664 (1840–3855)	1710 (979–2985)^*^	—
		D30	100 (88.4–100)	100 (80.5–100)	100 (83.9–100)	100 (86.3–100)	87 (66.4–97.2)	1676 (1164–2414)	1199 (849–1692)	58 (35–96)
	Dose 2	D3	—	—	—	100 (83.2–100)	—	—	—	94 (54–162)^**^
		D7	—	—	—	—	100 (84.6–100)	—	—	259 (149–449)^***^
		D30	—	100 (78.2–100)	100 (82.4–100)	100 (85.8–100)	100 (85.2–100)	—	181 (108–303)	188 (132–266)
W	Booster/Dose 1	Pre	75 (56.6–88.5)	70 (47.1–86.8)	87 (66.4–97.2)	44 (24.4–65.1)	64 (40.7–82.8)	28 (15–53)	25 (15–40)	8.37 (4.78–15)
		D3	76 (54.9–90.6)	73 (49.8–89.3)	—	—	—	19 (8.77–41)	20 (8.11–51)^*^	—
		D7	100 (86.3–100)	—	100 (79.4–100)	—	—	1285 (900–1833)	1693 (924–3103)^*^	—
		D30	100 (87.7–100)	100 (80.5–100)	100 (83.9–100)	100 (84.6–100)	92 (73.0–99.0)	1823 (1163–2856)	2259 (1678–3041)	117 (83–166)
	Dose 2	D3	—	—	—	100 (82.4–100)	—	—	—	180 (107–305)^**^
		D7	—	—	—	—	100 (83.2–100)	—	—	188 (129–272)^***^
		D30	—	100 (78.2–100)	100 (80.5–100)	100 (83.2–100)	100 (83.9–100)	—	85 (46–155)	168 (129–218)
Y	Booster/Dose 1	Pre	38 (21.1–56.3)	64 (40.7–82.8)	55 (32.2–75.6)	17 (5.0–38.8)	21 (7.1–42.2)	6.17 (3.00–13)	12 (6.29–22)	2.00 (1.40–2.85)
		D3	42 (24.5–60.9)	50 (28.2–71.8)	—	—	—	6.53 (3.05–14)	9.60 (3.56–26)^*^	—
		D7	100 (85.2–100)	—	100 (75.3–100)	—	—	722 (429–1216)	1133 (710–1809)^*^	—
		D30	100 (85.2–100)	100 (73.5–100)	100 (71.5–100)	91 (72–98.9)	78 (56.3–92.5)	779 (430–1413)	1692 (1206–2373)	85 (45–163)
	Dose 2	D3	—	—	—	96 (78.1–99.89)	—	—	—	109 (55–215)^**^
		D7	—	—	—	—	100 (83.2–100)	—	—	299 (180–495)^***^
		D30	—	100 (73.5–100)	100 (78.2–100)	100 (85.2–100)	95 (75.1–99.87)	—	264 (117–598)	224 (147–343)

Footnote: hSBA, human serum bactericidal assay; CI, confidence interval; N, number of participants in a group; GMT, geometric mean titer; Pre, pre-vaccination; D3, 3 days after vaccine booster/dose 1 or 2; D7, 7 days after vaccine booster/dose 1 or 2; D30, 30 days after vaccine booster/dose 1 or 2;

* n=23,

** n=26,

*** n=24, maximum number of participants with results at day 3 and day 7 in subgroups VIa, VIb, VIIa and VIIb. A “–“ is placed in the cells where no analysis was planned for those specific time points.

**Table 3.** Percentages of participants with hSBA ≥5 and geometric mean hSBA titers (95% CI) against serogroup B strains at different timepoints – FAS Immunogenicity Kinetics.

			% of participants with hSBA ≥5 (95% CI)					GMT (95% CI)		
			Group III	Group VI		Group VII		Group III	Group VI	Group VII
				a	b	a	b			
Strains (antigens)	Timepoint		N=33	N=23	N=23	N=26	N=24	N=33	N=46	N=50
H44/76 (fHbp)	Booster/Dose 1	Pre	27 (13.3–45.5)	9 (1.1–28.0)	0 (0–14.8)	12 (2.4–30.2)	8 (1.0–27.0)	2.33 (1.57–3.45)	1.21 (1.03–1.42)	1.44 (1.14–1.80)
		D3	21 (9.0–38.9)	4 (0.11–21.9)	—	—	—	2.58 (1.78–3.75)	1.38 (1.05–1.81)^*^	—
		D7	94 (79.8–99.3)	—	14 (2.9–34.9)	—	—	64 (38–109)	1.84 (1.18–2.89)^*^	—
		D30	97 (84.2–99.92)	52 (30.6–73.2)	52 (30.6–73.2)	73 (52.2–88.4)	46 (25.6–67.2)	98 (57–170)	5.78 (3.59–9.31)	7.27 (4.53–12)
	Dose 2	D3	—	—	—	69 (48.2–85.7)	—	—	—	8.91 (4.62–17)^**^
		D7	—	—	—	—	96 (78.9–99.89)	—	—	35 (20–60)^***^
		D30	—	78 (56.3–92.5)	91 (72–98.9	96 (79.6–99.9)	79 (57.8–92.9)	—	16 (10–24)	32 (21–49)
M14459 (fHbp)	Booster/Dose 1	Pre	18 (7.0–35.5)	9 (1.1–28.0)	0 (0–14.8)	8 (0.9–25.1)	8 (1.0–27.0)	2.04 (1.46–2.83)	1.37 (1.12–1.69)	1.48 (1.18–1.85)
		D3	21 (9.0–38.9)	4 (0.1–21.9)	—	—	—	1.99 (1.44–2.75)	1.22 (0.96–1.54)^*^	—
		D7	79 (61.1–91.0)	—	14 (2.9–34.9)	—	—	17 (9.31–30)	1.52 (1.05–2.20)^*^	—
		D30	85 (68.1–94.9)	35 (16.4–57.3)	27 (10.7–50.2)	46 (26.6–66.6)	33 (15.6–55.3)	24 (14–42)	2.83 (1.88–4.24)	4.02 (2.67–6.06)
	Dose 2	D3	—	—	—	42 (23.4–63.1)	—	—	—	5.06 (2.96–8.66)^**^
		D7	—	—	—	—	71 (48.9–87.4)	—	—	9.16 (5.35–16)^***^
		D30	—	43 (23.2–65.5)	48 (26.8–69.4)	80 (59.3–93.2)	54 (32.8–74.4)	—	3.50 (2.33–5.26)	9.78 (6.42–15)
5/99 (NadA)	Booster/Dose 1	Pre	70 (51.3–84.4)	22 (7.5–43.7)	13 (2.8–33.6)	19 (6.6–39.4)	13 (2.7–32.4)	13 (7.29–22)	1.78 (1.36–2.34)	1.90 (1.42–2.53)
		D3	79 (61.1–91.0)	9 (1.1–28.0)	—	—	—	17 (9.56–31)	1.99 (1.36–2.92)^*^	—
		D7	100 (89.4–100)	—	9 (1.1–29.2)	—	—	1768 (1204–2596)	1.97 (0.88–4.42)^*^	—
		D30	100 (89.4–100)	78 (56.3–92.5)	78 (56.3–92.5)	85 (65.1–95.6)	79 (57.8–92.9)	1426 (940–2164)	19 (11–34)	25 (15–42)
	Dose 2	D3	—	—	—	85 (65.1–95.6)	—	—	—	40 (18–90)^***^
		D7	—	—	—	—	100 (85.8–100)	—	—	432 (273–683)^***^
		D30	—	100 (85.2–100)	100 (85.2–100)	100 (86.3–100)	100 (85.8–100)	—	110 (76–159)	256 (194–338)
M01-0240364 (NadA)	Booster/Dose 1	Pre	13 (3.6–29.8)	9 (1.1–29.2)	0 (0–15.4)	4 (0.1–19.6)	4 (0.1–21.1)	2.26 (1.26–4.08)	1.14 (0.97–1.35)	1.23 (1.02–1.47)
		D3	17 (5.6–34.7)	4 (0.1–21.9)	—	—	—	2.50 (1.25–5.02)	1.36 (1.02–1.82)^*^	—
		D7	93 (77.2–99.2)	—	10 (1.2–30.4)	—	—	525 (257–1074)	1.65 (0.79–3.43)^*^	—
		D30	97 (82.8–99.9)	29 (11.3–52.2)	22 (7.5–43.7)	33 (15.6–55.3)	24 (8.2–47.2)	601 (339–1064)	3.05 (1.77–5.26)	3.11 (1.76–5.51)
	Dose 2	D3	—	—	—	40 (21.1–61.3)		—	—	5.13 (2.17-12)^**^
		D7	—	—	—	—	82 (59.7–94.8)	—	—	75 (29–199)^***^
		D30	—	65 (42.7–83.6)	65 (42.7–83.6)	70 (47.1–86.8)	71 (48.9–87.4)	—	16 (8.77–31)	21 (11–38)
96217 (NadA)	Booster/Dose 1	Pre	61 (42.1–77.1)	22 (7.5–43.7)	22 (7.5–43.7)	42 (23.4–63.1)	29 (12.6–51.1)	16 (8.89–28)	2.35 (1.51–3.64)	4.08 (2.52–6.62)
		D3	70 (51.3–84.4)	39 (19.7–61.5)	—	—	—	1211 (823–1781)	3.77 (1.81–7.89)^*^	—
		D7	100 (89.4–100)	—	36 (17.2–59.3)	—	—	970 (687–1369)	4.20 (1.75–10)^*^	—
		D30	100 (89.4–100)	57 (34.5–76.8)	74 (51.6–89.8)	73(52.2–88.4)	75(53.3–90.2)	—	15 (7.83–27)	19 (11–33)
	Dose2	D3	—	—	—	77 (56.4–91.0)	—	—	—	29 (13–67)^**^
		D7	—	—	—	—	100 (85.8–100)	—	—	309 (203–469)^***^
		D30	—	100 (85.2–100)	100 (85.2–100)	100 (86.3–100)	100 (85.8–100)	—	63 (38–103)	169 (129–222)
M10713 (NHBA)	Booster/Dose 1	Pre	48 (30.8–66.5)	48 (26.8–69.4)	17 (5.0–38.8)	38 (20.2–59.4)	38 (18.8–59.4)	3.66 (2.38–5.62)	2.56 (1.74–3.75 )	3.10 (2.16–4.45)
		D3	56 (37.7–73.6)	39 (19.7–61.5)	—	—	—	4.16 (2.70–6.41)	3.73 (2.10-6.65)^*^	—
		D7	79 (61.1–91.0)	—	32 (13.9–54.9)	—	—	15 (9.26–23)	2.03 (1.30–3.17)^*^	—
		D30	85 (68.1–94.9)	70 (47.1–86.8)	35 (16.4–57.3)	62 (40.6–79.8)	54 (32.8–74.4)	20 (13–31)	5.51 (3.45–8.79)	7.70 (5.04–12)
	Dose 2	D3	—	—	—	57 (34.5–76.8)	—	—	—	6.70 (3.37–13)^***^
		D7	—	—	—	—	67 (44.7–84.4)	—	—	8.94 (4.91–16)^***^
		D30	—	65 (42.7–83.6)	43 (23.2–65.5)	68 (46.5–85.1)	63 (40.6–81.2)	—	2.70 (1.94–3.77)	8.73 (5.80–13)
NZ98/254 (PorA)	Booster/Dose 1	Pre	15 (5.1–31.9)	4 (0.11–21.9)	0 (0–14.8)	8 (0.9–25.1)	4 (0.11–21.1)	1.81 (1.30–2.51)	1.35 (1.15–1.59)	1.45 (1.18–1.77)
		D3	12 (3.4–28.2)	13 (2.8–33.6)	—	—	—	1.90 (1.41–2.57)	1.89 (1.38–2.59)^**^	—
		D7	67 (48.2–82)	—	9 (1.1–29.2)	—	—	8.19 (5.45–12)	1.52 (1.12–2.06)^*^	—
		D30	82 (64.5–93.0)	48 (26.8–69.4)	26 (10.2–48.4)	58 (36.9–76.6)	46 (25.6–67.2)	13 (8.50–18)	3.57 (2.37–5.38)	5.75 (3.95–8.37)
	Dose 2	D3	—	—	—	50 (29.9–70.1)	—	—	—	6.93 (4.02–12)^**^
		D7	—	—	—	—	75 (53.3–90.2)	—	—	7.92 (5.03–12)^***^
		D30	—	52 (30.6–73.2)	39 (19.7–61.5)	72 (50.6–87.9)	50 (29.1–70.9)	9.15 (4.78–18)	4.61 (3.22–6.60)	7.50 (5.12–11)

hSBA, human serum bactericidal assay; CI, confidence interval; FAS, full analysis set; N, number of participants in a group; GMT, geometric mean titer; fHbp, factor H binding protein; NadA, *Neisseria* adhesin A; NHBA, Neisserial heparin-binding antigen; PorA, porin A. Pre, pre-vaccination; D3, 3 days after vaccine booster/dose 1 or 2; D7, 7 days after vaccine booster/dose 1 or 2; D30, 30 days after vaccine booster/dose 1 or 2;

* n=23,

** n=26,

*** n=24, maximum number of participants with results at day 3 and day 7 in subgroups VIa, VIb, VIIa and VIIb. A “–“ is placed in the cells where no analysis was planned for those specific time points.

In primed Groups III and VI, all participants achieved hSBA titers ≥8 against serogroups A, C, W and Y by 7 days after booster/dose 1, with the exception of the participants in Group VI against serogroup A, who achieved it 30 days after dose 1 (Table 2). In Group VII, for serogroups C and W, all participants achieved hSBA titers ≥8 at day 3 post-dose 2, and almost all participants achieved hSBA ≥8 against serogroups A and Y by the end of the study (Table 2). Two participants in Group VII who, by day 7 post-dose 2, had achieved hSBA titers ≥8 against serogroup A, and 1 who had achieved an hSBA titer ≥8 against serogroup Y no longer had hSBA titers ≥8 by day 30 post-dose 2 (Table 2).

For the primed Groups III and VI, hSBA GMTs against A, C, W and Y serogroups substantially increased at day 7 after booster/dose 1 with GMRs between 61 and 164 for Group III and 17 and 491 for Group VI. Thirty days post-booster/dose 1, GMTs increased compared to day 7, with the exception of serogroup A GMTs in Group III and serogroup C GMTs in Group III and VI, which moderately decreased (Table 2). For the non-primed Group VII, at day 30 post-dose 2, antibody GMTs against serogroup C moderately decreased compared to day 7, whereas GMTs for serogroups A, W and Y continued to increase (Table 2).

The immune response against serogroup B test strains did not follow a uniform pattern. By day 7 post-booster, between 67% and 100% of serogroup B-primed participants in Group III had achieved hSBA titers ≥5 against serogroup B test strains, and by day 30 post-booster this had increased to at least 82% (Table 3). In the non-primed participants in Groups VI and VII, the immune response after the first dose of MenABCWY+OMV varied widely, with few participants showing hSBA titers ≥5 7 days post-dose 1 (range 9%–36%) in Group VI. By day 30 post-dose 1, the percentages of participants with hSBA titer ≥5 ranged from 22% to 78% (Group VI) and from 24% to 85% (Group VII; Table 3). The percentages of participants with hSBA titers ≥5 continued to increase after the second dose, but by day 30 post-dose 2 all participants had hSBA titer ≥5 only for NadA test strain 5/99. Percentages against other strains were ranging from 39% to 78% (Group VI) and 50% to 96% (Group VII; Table 3).

After the booster vaccination in Group III, clear increases in serogroup B test strain GMTs were visible by day 7 post-vaccination, and antibody GMTs continued to increase to post-primary vaccination titers or higher after that (Table 3). In non-primed Groups VI and VII, antibody GMTs against all test strains were low after the first vaccination (2.83–25). In Group VII, GMTs started to increase after day 3 post-dose 2 (5.06–40), resulting in higher titers (7.5–256) by day 30 post-dose 2 for all strains compared to Group VI (2.7–110) at the same time point (Table 3).

#### Over-time response against serogroup B test strains

An over-time assessment of the vaccine responses from the primary and first extension study of participants who received 2 doses of MenABCWY+OMV (Group III) showed that percentages of participants achieving hSBA titers ≥5 post-booster were comparable with or higher than post-primary vaccination against all serogroup B strains. Although hSBA GMTs waned after primary vaccination, pre-booster GMTs were higher compared with baseline values and highly increased again after booster administration (**data not shown**).

### Safety and reactogenicity

Out of the 129 participants vaccinated with MenABCWY+OMV in Groups III, VI, and VII, 122 (95%) reported at least 1 solicited adverse event (AE) after booster/dose 1. Out of the 96 participants in Groups VI and VII who received a second vaccination, 80 (84%) reported a solicited AE. Frequency of both injection site and systemic reactions appeared to be reduced after the second vaccination. There were no apparent differences in solicited AEs between groups (Table 4).

**Table 4.** Number (percentage) of participants with solicited, unsolicited and serious adverse events after each MenABCWY+OMV dose.

	Group III	Group VI		Group VII	
	Booster dose	1^st^ dose	2^nd^ dose	1^st^ dose	2^nd^ dose
	N=33	N=46	N=46	N=50	N=50
Local symptoms					
Any	31 (94%)	41 (89%)	35 (78%)	47 (94%)	41 (82%)
Pain					
Any	31 (94%)	41 (89%)	34 (76%)	47 (94%)	41 (82%)
Severe	6 (18%)	8 (17%)	2 (4%)	6 (12%)	3 (6%)
Erythema					
Any	8 (24%)	7 (16%)	6 (13%)	8 (16%)	7 (14%)
Severe	1 (3%)	1 (2%)	0	0	0
Induration					
Any	12 (36%)	7 (15%)	9 (20%)	13 (26%)	8(16%)
Severe	0	0	0	1 (2%)	0
General symptoms					
Any	24 (73%)	35 (76%)	22 (49%)	37 (74%)	28 (56%)
Chills					
Any	5 (15%)	11 (24%)	5 (11%)	11 (22%)	9 (18%)
Severe	1 (3%)	0	1 (2%)	0	1 (2%)
Loss of appetite					
Any	5 (15%)	9 (20%)	3 (7%)	9 (18%)	5 (10%)
Severe	0	0	0	1 (2%)	2 (4%)
Headache					
Any	19 (58%)	20 (43%)	11 (25%)	23 (46%)	18 (36%)
Severe	0	1 (2%)	2 (5%)	3 (6%)	4 (8%)
Fatigue					
Any	14 (42%)	19 (42%)	10 (23%)	24 (48%)	12 (24%)
Severe	0	1 (2%)	1 (2%)	0	1 (2%)
Myalgia					
Any	18 (55%)	22 (48%)	12 (27%)	23 (46%)	14 (28%)
Severe	0	0	1 (2%)	1 (2%)	2 (4%)
Arthralgia					
Any	8 (24%)	8 (17%)	4 (9%)	15 (30%)	7 (14%)
Severe	0	0	1 (2%)	0	1 (2%)
Nausea					
Any	4 (12%)	10 (22%)	4 (9%)	13 (26%)	4 (8%)
Severe	0	1 (2%)	0	0	0
Fever					
Any(≥38°C)	0	4 (9%)	4 (9%)	8 (16%)	2 (4%)
Severe(≥40°C)	0	0	0	0	0
Unsolicited symptoms	11 (33%)	21 (46%)		20 (40%)	
Possibly related unsolicited AEs	3 (9%)	11 (24%)		8 (16%)	
Medically-attended AEs	2 (6%)	6 (13%)		5 (10%)	
SAEs	0	0	0		

Footnote: N, maximum number of participants with available results; AE, adverse event; SAE, serious adverse event.

The most commonly reported injection site reaction was pain and the most commonly reported systemic reactions were myalgia and headache (Table 4).

Across vaccine groups, 52 participants (40%) reported at least 1 unsolicited AE (Table 4). No differences were apparent between groups. Injection site induration continuing after 7 days of the vaccination was the most frequently reported unsolicited AE (Group III: 6%; Group VI: 15%; Group VII: 14%).

Unsolicited AEs that study investigators considered to be potentially related to the study vaccine were reported by 22 (17%) participants; injection site induration was the most frequently reported (16 [12%] participants). AEs requiring medical attention were reported for 13 participants (10%). One participant in Group VI was withdrawn from the study due to presyncope at day 16 post-dose 1. Most unsolicited AEs were mild to moderate in intensity, and were resolved before the end of the study.

No death or serious AEs (SAEs) were reported during this study.

## Discussion

In this open-label extension study, we report for the first time that antibodies against A, C, W and Y serogroups persist above pre-vaccination levels for up to 4 years after 2 doses of the MenABCWY+OMV vaccine administered to healthy adolescents. Antibody concentrations against serogroup B test strains waned over time, but administration of a booster dose resulted in substantial increases in antibody concentrations against all test strains. In addition, our study results indicate that a MenABCWY+OMV booster administered more than 4 years after primary vaccination boosts the immune response to serogroups A, C, W and Y both in adolescents and young adults who previously received the pentavalent MenABCWY+OMV vaccine and in adolescents and young adults who received quadrivalent MenACWY-CRM.

Antibody persistence against serogroups A, C, W and Y was observed for up to 4 years post-vaccination with 2 doses of MenABCWY+OMV or 1 dose of MenACWY-CRM with levels comparable between both groups. The antibody persistence observed in MenACWY-CRM primed participants (Group VI) compared with those observed in other studies in adolescents at 3 and 5 years after 1 dose of MenACWY-CRM shows similar percentages of participants with hSBA titres ≥8 against serogroups A, C and W, and lower percentages against serogroup Y.^31^^,^^32^ However any comparison should be made with caution as different assays were used for sample testing and these studies were conducted in different countries with different prevalence of meningococcal strains.

A decrease in antibody concentrations against serogroup B test strains was observed 4 years post vaccination with 2 doses of MenABCWY+OMV. This extends findings from the first extension study^29^ that shows antibody persistence for up to 12 months against serogroups A, C, W and Y and the serogroup B 5/99 test strain after 2 doses of the vaccine.^29^ At one month, 10 months or 4 years post-vaccination, the immune responses against serogroup B strains were not uniform, with more participants retaining antibodies against strains H44/76 (fHbp) and 5/99 (NadA) than against NZ98/254 (PorA). These antibody persistence results against serogroups B strains after 2-dose MenABCWY+OMV administration appear to be in line with the 4-year persistence data after 2-dose 4CMenB administration.^33^ However, any comparisons should be made with caution as the assays used in the two studies were different. The immune response observed in vaccine-naïve participants (Group VII) against serogroups A, C, W, Y and B in this extension study are similar to the responses observed in MenABCWY+OMV recipients in the primary study.^28^

A single booster dose in participants who previously received 2 doses of the MenABCWY+OMV vaccine induced an anamnestic immune response, with antibody titers several-fold higher than post-primary responses for most serogroups and strains. Indeed, antibody titers above putative protective cut off values were seen as early as 7 days after the booster, indicating induction of immune memory by the priming schedule, which may be important to control disease outbreaks. While most adolescents previously exposed to the serogroup B antigens achieved a full antibody response against serogroup B test strains, the response against these strains varied widely in participants who were naïve to serogroup B vaccines. Although previous meningococcal vaccination resulted in retention of specific bactericidal antibodies for up to 4 years in some of the adolescents, antibody titers waned below putative protective levels, as early as 10 months after priming^29^ with further declines by 4 years. A booster dose rapidly re-established protective antibody levels in most adolescents, with clear evidence of an anamnestic response. Therefore, immune memory alone may not offer adequate protection, and a booster dose may prove essential to ensure continued protection of previously vaccinated adolescents against meningococcal disease. However, since persistence of bactericidal antibodies varies by serogroup B strain, the need and timing of a booster dose may also theoretically vary by strain, and be influenced by prevailing epidemiology, which may warrant further investigation.

Our evaluation of vaccine safety indicates a reactogenicity profile similar to that previously reported.^28^^,^^29^ The frequency and severity of solicited reactions observed after vaccination with MenABCWY+OMV were similar to reactions observed after 4CMenB vaccination,^27^^,^^32^ but higher than those seen after vaccination with MenACWY-CRM, as expected.^34^

This study had a few limitations. Enrollment in the study was limited to the eligible participants who received vaccination in two of the groups in the primary trial (Group III and Group VI) and were not vaccinated in the first extension.^28^^,^^29^ Since the enrollment was based on the former participants' willingness, in this second extension we could not ensure equal distribution of participants across groups and blood draw subsets. As a result, study objectives were descriptive in nature and meaningful comparisons could not be made across groups. Furthermore, the assay format used to test samples was different from that historically used for both the MenACWY and the MenB vaccine trials, limiting comparability with previous data. Another limitation that should be considered is that an alternative dosing schedule for MenABCWY+OMV (2 doses, 1 month apart) has been used in this trial, while the schedule in this age group, as determined from phase 1 and early phase 2 trials,^28^^,^^29^ is 2 doses, 2 months apart. Finally, the results of this trial may not be applicable for other serogroup B-containing vaccines, ACWY conjugate vaccines, or other investigational pentavalent vaccines, and observations cannot be extrapolated to other meningococcal vaccines.

In conclusion, we have observed that an investigational pentavalent meningococcal vaccine induced long-term persistence of antibodies against all 5 serogroups, although to varying degrees, when administered to adolescents as a 2-dose schedule. Furthermore, a booster dose administered at 4 years post-primary vaccination, was able to induce a rapid and anamnestic response, both in participants primed with the same vaccine and those previously exposed to a different (quadrivalent) meningococcal vaccine. These promising findings suggest that a combination meningococcal vaccine, offering broad protection against the most prevalent disease-causing serogroups, may be suitable both in the context of simplification of vaccination schedules and, potentially, in outbreak management.

## Methods

### Study participants, design, and objectives

This was a phase II, open-label, controlled, multicenter extension study (NCT02451514) conducted between June 2015 and December 2015 in 9 centers in Chile, Colombia and Panama.

This extension study followed 2 groups (Group III and VI) from the primary study conducted between December 2010 and July 2011 (NCT01210885)^28^ and the first extension conducted between July 2011 and July 2012 (NCT01367158).^29^

This study was conducted in accordance with Good Clinical Practice and the Declaration of Helsinki. The protocol and informed consent forms were reviewed and approved by an Independent Ethics Committee or Institutional Review Board before the study start. This study is registered at www.clinicaltrials.gov NCT02451514. A summary of the protocol is available at http://www.gsk-clinicalstudyregister.com (study ID 205213). Written informed consent/assent was given by participant(s) or their parent(s)/legal guardian(s) if the participant(s) were under 18 years.

#### Participants

Participants were adolescents and young adults between 15 and 23 years of age and, who received either 2 doses of MenABCWY+OMV (Group III) or a single dose of MenACWY-CRM (Group VI) in the primary study conducted approximately 48 to 56 months before this study.^28^

During a first extension study conducted 12 months after the primary, study participants received a tetanus-diphtheria-pertussis vaccination.^29^ In addition to participants previously exposed to meningococcal vaccines, we also included a newly-recruited group of participants naïve to all meningococcal vaccines (Group VII).

A complete list of inclusion and exclusion criteria is provided in **Supplementary Material 1**.

#### Design

Study design is presented in Fig. 2.

At 48 to 56 months following the primary study, participants vaccinated with 2 doses of MenABCWY+OMV in the primary study received a booster dose of the MenABCWY+OMV vaccine (Group III). Participants who in the primary study received 1 dose of MenACWY-CRM and 1 dose of placebo (Group VI), and the newly-recruited meningococcal-vaccine-naïve participants (Group VII) received 2 doses of the MenABCWY+OMV vaccine 30 days apart.

In participants in Group III, blood was collected prior to vaccination and at days 3, 7, and 30 after vaccination. Groups VI and VII were split 1:1 and blood was collected prior to vaccination and at days 3 and 30 (subgroups a) or days 7 and 30 (subgroups b) after each vaccination (Fig. 2).

#### Study objectives

The study evaluated the immune response and safety after 1 or 2 doses of the MenABCWY+OMV vaccine. The primary objectives were the assessment of 4-year antibody persistence against *N. meningitidis* serogroups A, C, W and Y and serogroup B test strains in meningococcal-vaccine primed groups (Groups III and VI) and evaluated the immune response 30 days after a single dose of the MenABCWY+OMV vaccine in all groups.

The secondary objectives were the assessment of the immune response at days 3, 7 and 30 after vaccination with the MenABCWY+OMV vaccine in participants primed with 2 doses of MenABCWY+OMV (Group III); and the immune response at days 3, 7 and 30 after each vaccination with the MenABCWY+OMV vaccine in participants who received 1 dose of MenABCWY+OMV vaccine and one dose of placebo (Group VI) and vaccine-naïve participants (Group VII). Safety and reactogenicity of the MenABCWY+OMV vaccine was also assessed in all participants.

### Vaccine

Participants received 1 or 2 doses of the MenABCWY+OMV investigational vaccine, lot number JA145001.

MenABCWY+OMV vaccine was reconstituted of lyophilized powder containing the capsular oligosaccharide of serogroups ACWY conjugated to a carrier protein CRM_197_ combined with recombinant 4CMenB+OMV that contains recombinant proteins of *N meningitidis* (936-741, 287-953 and 961c) serogroup B and OMV from serogroup B strain NZ98/254 adsorbed onto aluminum hydroxide.^28^

The vaccine was administered as a 0.5 mL dose injected intramuscularly.

### Immunogenicity assessment

Antibody titers were measured using hSBA.^35^

Immunogenicity for *N. meningitidis* serogroups A, C, W, Y and serogroup B test strains (H44/76, 5/99, M14459, M10713, M01-0240364, NZ98/254 and 96217) was measured by high throughput serum bactericidal assay using human complement.^35^

Post-vaccination, an hSBA titer ≥4 is accepted as the serologic correlate of protection against serogroup C^36^^,^^37^ and, by extrapolation, for other meningococcal serogroups. However, more conservative cut-offs, of ≥8 for serogroups A, C, W and Y and ≥5 for serogroup B, were used in this study. The immunogenicity endpoints related to percentages of participants with hSBA titers ≥ lower limit of quantitation (LLOQ) were not independently assessed for this study. The HT-hSBA assay format used for sample testing is associated with LLOQ values of 8 for the ACWY serogroups and 5 for serogroup B strains, and these endpoints have been assessed and reported.

A detailed description of immunogenicity assessment is presented in **Supplementary Material 2**.

### Safety assessment

Solicited local (pain, erythema and induration) and systemic (fatigue, headache, myalgia, arthralgia, loss of appetite, nausea, chills, fever [oral temperature ≥38°C]) AEs occurring within 7 days (day 1–7) after vaccination and unsolicited AEs occurring within 30 days (day 1–30) after vaccination were recorded by participant or participant's parent/legal guardian using diary cards.

Medically-attended AEs, AEs leading to withdrawal and SAEs were collected during the entire study period. These data were captured interviewing the participants and/or participants' parents/guardian during the site visits and by review of available medical records.

The relationship of the study treatment to an AE and its severity was determined by the investigator.

### Statistical analysis

Determination of the sample size is presented in **Supplementary Material 3**.

All participants who were exposed to the study vaccine were included in the safety analysis.

Four-year persistence of *N. meningitidis* serogroups A, C, W and Y and serogroup B test strains in meningococcal-vaccine primed groups (Groups III and VI) was evaluated in the full analysis set (FAS) for Persistence, consisting of all participants with at least one evaluable serum sample at baseline for which an hSBA result was available for at least 1 strain. For these participants, we evaluated persistence in terms of hSBA thresholds (percentage of participants presenting with hSBA≥8 [against serogroups ACWY] and ≥5 [against serogroup B test strains] and 95% Clopper-Pearson CIs) and antibody GMTs and associated 95% CIs.

The primary and secondary objectives for the assessment of immune response were evaluated in the FAS for Immunogenicity, consisting of all participants who received one study vaccine and provided at least one evaluable serum sample post-vaccination for which an hSBA result was available for at least 1 strain. We evaluated these endpoints in all study groups in terms of hSBA thresholds and antibody GMTs. No inferential statistics were done, and all results are descriptive in nature.

GMTs were provided along with the associated 95% CIs, they were computed by exponentiating (base 10) the unadjusted means and the lower and upper limits of the 95% CIs of the log transformed titers (base 10), for each study group and *N. meningitidis* serogroups A, C, W, and Y and serogroup B test strains. In addition, we calculated GMRs over baseline at day 61 after the first dose of MenABCWY+OMV for groups VI and VII.

## Trademarks

*Bexsero* and *Menveo* are trademarks owned by GSK groups of companies.

## Supplementary Material

KHVI_A_1457595_Supplemental.zip

## Abbreviations

AE: adverse event
CRM_197_: carrier protein cross reactive material 197
ELISA: enzyme-linked immunosorbent assay
FAS: full analysis set
fHbp: factor H binding protein
GMR: geometric mean ratio
GMT: geometric mean titer
hSBA: human serum bactericidal assay,
HT-hSBA: high throughput serum bactericidal assay using human complement
IMD: invasive meningococcal disease
LLOQ: lower limit of quantitation
MenABCWY+OMV: investigational vaccine containing MenACWY conjugated to a carrier protein CRM_197_ combined with recombinant proteins from serogroup B and outer membrane vesicle from New Zealand
MenACWY-CRM: meningococcal quadrivalent vaccine for serogroups A, C, W, and Y conjugated to a carrier protein CRM_197_
NadA: *Neisseria* adhesin A
NHBA: Neisserial heparin-binding antigen
OMV: outer membrane vesicles
PorA: porin A
SAE: serious adverse event.
4CMenB: 4-component serogroup B recombinant meningococcal vaccine

## References

[1] Rosenstein NE, Perkins BA, Stephens DS, Popovic T, Hughes JM Meningococcal Disease. N Engl J Med. 2001;344(18):1378–88. doi:10.1056/NEJM200105033441807. PMID:11333996. 11333996

[2] European Centre for Disease Prevention and Control Surveillance of invasive bacterial diseases in Europe 2007. [accessed 214, 2018]. http://www.ecdc.europa. eu/en/publications/Publications/101011_SUR_Surveillance_of_invasive_bacterial_diseases_in_Europe_2007.pdf.

[3] Pace D, Pollard AJ Meningococcal disease: clinical presentation and sequelae. Vaccine. 2012;30(Suppl 2):B3–9. doi:10.1016/j.vaccine.2011.12.062. PMID:22607896. 22607896

[4] Erlich KS, Congeni BL Importance of circulating antibodies in protection against meningococcal disease. Hum Vacc Immunotherapeutics. 2012;8(8):1029–35. doi:10.4161/hv.20473. PMC3551872 22854672

[5] Pelton SI The global evolution of meningococcal epidemiology following the introduction of meningococcal vaccines. J Adolesc Health. 2016;59(2 Suppl):S3–S11. doi:10.1016/j.jadohealth.2016.04.012. PMID:27449148. 27449148

[6] World Health Organization Meningococcal meningitis. Fact sheet no 141; 2015 [accessed 20171121]. http://www.who.int/mediacentre/factsheets/fs141/en/.

[7] Abad R, Lopez EL, Debbag R, Vazquez JA Serogroup W meningococcal disease: global spread and current affect on the Southern Cone in Latin America. Epidemiol Infect. 2014;142(12):2461–70. doi:10.1017/S0950268814001149. PMID:24831052. 24831052 PMC9151320

[8] Gianchecchi E, Torelli A, Piccini G, Piccirella S, Montomoli E *Neisseria meningitidis* infection: who, when and where? Expert Rev Anti Infect Ther. 2015;13(10):1249–63. doi:10.1586/14787210.2015.1070096. PMID:26190347. 26190347

[9] Jafri RZ, Ali A, Messonnier NE, Tevi-Benissan C, Durrheim D, Eskola J, Fermon F, Klugman KP, Ramsay M, Sow S, et al. Global epidemiology of invasive meningococcal disease. Population Health Metrics. 2013;11(1):17. doi:10.1186/1478-7954-11-17. PMID:24016339. 24016339 PMC3848799

[10] Safadi MA, Cintra OA Epidemiology of meningococcal disease in Latin America: current situation and opportunities for prevention. Neurol Res. 2010;32(3):263–71. doi:10.1179/016164110X12644252260754. PMID:20406604. 20406604

[11] Safadi MA, de los Monteros LE, Lopez EL, Saez-Llorens X, Lemos AP, Moreno-Espinosa S, Ayala SG, Torres JP, de Moraes JC, Vazquez JA The current situation of meningococcal disease in Latin America and recommendations for a new case definition from the Global Meningococcal Initiative. Expert Rev Vaccines. 2013;12(8):903–15. doi:10.1586/14760584.2013.814879. PMID:23909747. 23909747

[12] Macneil JR, Thomas JD, Cohn AC Meningococcal disease: shifting epidemiology and genetic mechanisms that may contribute to serogroup C virulence. Curr Infect Dis Rep. 2011;13(4):374–9. doi:10.1007/s11908-011-0195-7. PMID:21603878. 21603878

[13] Miller E, Salisbury D, Ramsay M Planning, registration, and implementation of an immunisation campaign against meningococcal serogroup C disease in the UK: a success story. Vaccine. 2001;20:s58–67. doi:10.1016/S0264-410X(01)00299-7. PMID:11587814. 11587814

[14] Borrow R, Alarcon P, Carlos J, Caugant DA, Christensen H, Debbag R, De Wals P, Echaniz-Aviles G, Findlow J, Head C, et al. The Global Meningococcal Initiative: global epidemiology, the impact of vaccines on meningococcal disease and the importance of herd protection. Expert Rev Vaccines. 2017;16(4):313–28. doi:10.1080/14760584.2017.1258308. PMID:27820969. 27820969

[15] Ministry of Health Vaccination in special populations; 2014 [accessed 20171127]. http://www.msal.gob.ar/images/stories/bes/graficos/0000000442cnt-2014-04_lineamientos-huespedes-especiales.pdf.

[16] Ministry of Health National immunisation calendar; 2017 [accessed 20171127]. http://www.msal.gob.ar/images/stories/ryc/graficos/0000001013cnt-2017-01-01_calendario-vacunacion.pdf.

[17] Ministry of Health Government of Chile. Vaccination calendar 2017. [accessed 20171127]. http://www.enfermeriaaps.com/portal/wp-content/uploads/2017/03/Calendario-vacunacion-Chile-2017.pdf.

[18] World Health Organization WHO vaccine-preventable diseases: monitoring system. 2017 global summary; [accessed 20171011]. http://apps.who.int/immunization_monitoring/globalsummary/schedules.

[19] Watson PS, Turner DP Clinical experience with the meningococcal B vaccine, Bexsero((R)): Prospects for reducing the burden of meningococcal serogroup B disease. Vaccine. 2016;34(7):875–80. doi:10.1016/j.vaccine.2015.11.057. PMID:26686570. 26686570

[20] Giuliani MM, Biolchi A, Serruto D, Ferlicca F, Vienken K, Oster P, Rappuoli R, Pizza M, Donnelly J Measuring antigen-specific bactericidal responses to a multicomponent vaccine against serogroup B meningococcus. Vaccine. 2010;28(31):5023–30. doi:10.1016/j.vaccine.2010.05.014. PMID:20493284. 20493284

[21] Toneatto D, Pizza M, Masignani V, Rappuoli R Emerging experience with meningococcal serogroup B protein vaccines. Expert Rev Vaccines. 2017;16(5):433–51. doi:10.1080/14760584.2017.1308828. PMID:28375029. 28375029

[22] Findlow J, Borrow R, Snape MD, Dawson T, Holland A, John TM, Evans A, Telford KL, Ypma E, Toneatto D, et al. Multicenter, open-label, randomized phase II controlled trial of an investigational recombinant meningococcal serogroup B vaccine with and without outer membrane vesicles, administered in infancy. Clin Infect Dis. 2010;51(10):1127–37. doi:10.1086/656741. PMID:20954968. 20954968

[23] Santolaya ME, O'Ryan ML, Valenzuela MT, Prado V, Vergara R, Munoz A, Toneatto D, Grana G, Wang H, Clemens R, et al. Immunogenicity and tolerability of a multicomponent meningococcal serogroup B (4CMenB) vaccine in healthy adolescents in Chile: a phase 2b/3 randomised, observer-blind, placebo-controlled study. Lancet. 2012;379(9816):617–24. doi:10.1016/S0140-6736(11)61713-3. PMID:22260988. 22260988

[24] Snape MD, Dawson T, Oster P, Evans A, John TM, Ohene-Kena B, Findlow J, Yu LM, Borrow R, Ypma E, et al. Immunogenicity of two investigational serogroup B meningococcal vaccines in the first year of life: a randomized comparative trial. Pediatr Infect Dis J. 2010;29(11):e71–9. doi:10.1097/INF.0b013e3181f59f6d. PMID:20844462. 20844462

[25] Vesikari T, Esposito S, Prymula R, Ypma E, Kohl I, Toneatto D, Dull P, Kimura A E. U. Meningococcal B Infant Vaccine Study group. Immunogenicity and safety of an investigational multicomponent, recombinant, meningococcal serogroup B vaccine (4CMenB) administered concomitantly with routine infant and child vaccinations: results of two randomised trials. Lancet. 2013;381(9869):825–35. doi:10.1016/S0140-6736(12)61961-8. PMID:23324563. 23324563

[26] Findlow J, Bai X, Findlow H, Newton E, Kaczmarski E, Miller E, Borrow R Safety and immunogenicity of a four-component meningococcal group B vaccine (4CMenB) and a quadrivalent meningococcal group ACWY conjugate vaccine administered concomitantly in healthy laboratory workers. Vaccine. 2015;33(29):3322–30. doi:10.1016/j.vaccine.2015.05.027. PMID:26025807. 26025807

[27] Kimura A, Toneatto D, Kleinschmidt A, Wang H, Dull P Immunogenicity and safety of a multicomponent meningococcal serogroup B vaccine and a quadrivalent meningococcal CRM197 conjugate vaccine against serogroups A, C, W-135, and Y in adults who are at increased risk for occupational exposure to meningococcal isolates. Clin Vaccine Immunol. 2011;18(3):483–6. doi:10.1128/CVI.00304-10. PMID:21177912. 21177912 PMC3067382

[28] Sáez-Llorens X, Aguilera Vaca DC, Abarca K, Maho E, Grana MG, Heijnen E, Smolenov I, Dull PM Immunogenicity and safety of investigational vaccine formulations against meningococcal serogroups A, B, C, W, and Y in healthy adolescents. Hum Vaccin Immunother. 2015;11(6):1507–17. doi:10.1080/21645515.2015.1029686. PMID:25969894. 25969894 PMC4514249

[29] Sáez-Llorens X, Aguilera Vaca DC, Abarca K, Maho E, Han L, Smolenov I, Dull P Persistence of meningococcal antibodies and response to a third dose after a two-dose vaccination series with investigational MenABCWY vaccine formulations in adolescents. Pediatr Infect Dis J. 2015;34(10):e264–78. doi:10.1097/INF.0000000000000822. PMID:26135245. 26135245

[30] Baxter R, Reisinger K, Block SL, Izu A, Odrljin T, Dull P Antibody persistence and booster response of a quadrivalent meningococcal conjugate vaccine in adolescents. J Pediatr. 2014;164(6):1409–15 e4. doi:10.1016/j.jpeds.2014.02.025. PMID:24657122. 24657122

[31] Baxter R, Reisinger K, Block SL, Percell S, Odrljin T, Dull PM, Smolenov I Antibody persistence after primary and booster doses of a quadrivalent meningococcal conjugate vaccine in adolescents. Pediatr Infect Dis J. 2014;33(11):1169–76. doi:10.1097/INF.0000000000000438. PMID:24911896. 24911896

[32] Jacobson RM, Jackson LA, Reisinger K, Izu A, Odrljin T, Dull PM Antibody persistence and response to a booster dose of a quadrivalent conjugate vaccine for meningococcal disease in adolescents. Pediatr Infect Dis J. 2013;32(4):e170–7. doi:10.1097/INF.0b013e318279ac38. PMID:23114372. 23114372

[33] Nolan T, Garfield H, Gupta A, Ferguson M, Marshall H, D'Agostino D, Toneatto D Persistence of bactericidal activity at 4 years after 2 primary doses of a recombinant, 4-component, meningococcal serogroup B vaccine (4CMenB) and response to a booster dose in adolescents and young adults. Open Forum Infect Dis. 2017;4(Suppl 1):S322 IDWeek 2017. doi:10.1093/ofid/ofx163.757.

[34] Toneatto D, Ismaili S, Ypma E, Vienken K, Oster P, Dull P The first use of an investigational multicomponent meningococcal serogroup B vaccine (4CMenB) in humans. Hum Vaccin. 2011;7(6):646–53. doi:10.4161/hv.7.6.15482. PMID:21904120. 21904120

[35] Mak PA, Santos GF, Masterman KA, Janes J, Wacknov B, Vienken K, Giuliani M, Herman AE, Cooke M, Mbow ML, et al. Development of an automated, high-throughput bactericidal assay that measures cellular respiration as a survival readout for *Neisseria meningitidis*. Clin Vaccine Immunol. 2011;18(8):1252–60. doi:10.1128/CVI.05028-11. PMID:21715580. 21715580 PMC3147359

[36] Goldschneider I, Gotschlich EC, Artenstein MS Human immunity to the meningococcus : I. The role of the humoral antibodies. J Exp Med. 1969;129(6):1307–26. doi:10.1084/jem.129.6.1307. PMID:4977280. 4977280 PMC2138650

[37] Goldschneider I, Gotschlich EC, Artenstein MS Human immunity to the meningococcus. II. Development of natural immunity. J Exp Med. 1969;129(6):1327–48. doi:10.1084/jem.129.6.1327. PMID:4977281. 4977281 PMC2138665

