# Supplementary material for: Four-year antibody persistence and response to a booster dose of a pentavalent MenABCWY vaccine administered to healthy adolescents and young adults
Source: Hum Vaccin Immunother. 2018 May 9;14(5):1161–74. doi: 10.1080/21645515.2018.1457595 (PMC5989907; doi:10.1080/21645515.2018.1457595)
Supplement: KHVI_A_1457595_Supplemental.zip [file khvi-14-05-1457595-s001.zip › KHVI_A_1457595_Supplemental Table.docx]

**Table S1.** Geometric mean concentrations measured by ELISA at different timepoints in the primary study, at year 1 and at year 4 -FAS Persistence

|  |  |  | Group III | | Group VI | | | Group VII | |
| --- | --- | --- | --- | --- | --- | --- | --- | --- | --- |
| Serogroup | Study | Timepoint | N | GMC (95%CI) | N | GMC (95%CI) | | N | GMC (95%CI) |
| A | Parent study | Baseline | 9 | 2.48 (0.91-6.82) | 18 | 1.92 (1.20-3.07) | |  |  |
|  |  | 1MPD1 | 13 | 23 (9.46-56) | 18 | 65 (28-151) | |  |  |
|  |  | 1MPD2 | 12 | 25 (11-60) |  |  | |  |  |
|  | Extension 1 | 1YP | 18 | 10 (6.04-18) | 18 | 22 (12-40) | |  |  |
|  | Extension 2 | 4YP | 19 | 7.34 (4.04-13) | 24 | 14 (8.65-24) | | 29 | 2.20 (1.57-3.08) |
| C | Parent study | Baseline | 9 | 0.28 (0.13-0.58) | 18 | 0.22 (0.15-0.34) |  |  |  |
|  |  | 1MPD1 | 13 | 17 (6.77-41) | 18 | 15 (7.54-31) |  |  |  |
|  |  | 1MPD2 | 12 | 10 (4.92-20) |  |  |  |  |  |
|  | Extension 1 | 1YP | 18 | 4.51 (2.13-9.55) | 18 | 2.63 (1.33-5.22) |  |  |  |
|  | Extension 2 | 4YP | 19 | 2.71 (1.37-5.39) | 24 | 1.64 (0.93-2.90) |  | 29 | 0.16 (0.10-0.26) |
| W | Parent study | Baseline | 9 | 0.28 (0.14-0.55) | 16 | 0.27 (0.17-0.43) |  |  |  |
|  |  | 1MPD1 | 13 | 17 (8.88-34) | 18 | 32 (16-64) |  |  |  |
|  |  | 1MPD2 | 12 | 16 (8.98-30) |  |  |  |  |  |
|  | Extension 1 | 1YP | 18 | 6.2 (3.75-10) | 18 | 10 (5.49-18) |  |  |  |
|  | Extension 2 | 4YP | 19 | 3.81 (2.38-6.09) | 24 | 4.81 (3.01-7.69) |  | 29 | 0.57 (0.45-0.73) |
| Y | Parent study | Baseline | 9 | 0.84 (0.46-1.55) | 18 | 0.66 (0.43-1.01) |  |  |  |
|  |  | 1MPD1 | 13 | 7.85 (4.72-13) | 18 | 21 (9.61-45) |  |  |  |
|  |  | 1MPD2 | 12 | 16 (9.78-25) |  |  |  |  |  |
|  | Extension 1 | 1YP | 18 | 7.63 (4.91-12) | 18 | 12 (6.79-20) |  |  |  |
|  | Extension 2 | 4YP | 19 | 5.67 (3.81-8.43) | 24 | 8.16 (5.20-13) |  | 29 | 2.77 (1.93-3.99) |

Footnote: ELISA, enzyme-linked immunosorbent assay; FAS, full analysis set; GMC, geometric mean concentration; N, number of participants with available results; Baseline, pre-vaccination in the primary study; 1MPD1, 1 month post-dose 1 in the primary study; 1MPD2, 1 month post-dose 2 in the primary study; 1YP, 1 year post-dose 1, extension study 1; 4YP, 4 years post-dose 1, pre-vaccination in the extension study 2. Group III, participants vaccinated in primary study with 2 doses of MenABCWY+OMV and in extension study 2 with a MenABCWY+OMV booster; Group VI, participants vaccinated in primary study with 1 dose of MenACWY-CRM and 1 dose of placebo and in extension study 2 with 2 doses of MenABCWY+OMV 1 month apart; Group VII, vaccine-naïve participants enrolled only in the extension study 2 and vaccinated with 2 doses of MenABCWY+OMV 1 month apart (here is presented only pre-vaccination time point).
