# Supplementary material for: Four-year antibody persistence and response to a booster dose of a pentavalent MenABCWY vaccine administered to healthy adolescents and young adults
Source: Hum Vaccin Immunother. 2018 May 9;14(5):1161–74. doi: 10.1080/21645515.2018.1457595 (PMC5989907; doi:10.1080/21645515.2018.1457595)
Supplement: KHVI_A_1457595_Supplemental.zip [file khvi-14-05-1457595-s001.zip › KHVI_A_1457595_Supplemental1.docx]

**Supplementary Material 1**

**Inclusion criteria:**

To be included in this study participants were:

- Individuals who received either 2 doses of MenABCWY+OMV or a single dose of MenACWY-CRM followed by a dose of placebo, with the last study vaccine given approximately 48 to 56 months before study extension 2, who received tetanus diphtheria and pertussis only in study extension 1 and who received no other meningococcal vaccines.

Or:

- Individuals who did not participate in the parent study and have not previously received any meningococcal vaccine and who /whose parent(s)/legal guardian(s) have given written informed consent/assent after the nature of the study has been explained according to local regulatory requirements, prior to study entry.
- Individuals who can comply with blood draws and follow-up.
- Males, female participants were not pregnant or breastfeeding and were using an effective birth control method which they have used for at least 30 days prior to study entry, and which they intend to use for at least 30 days after the last study vaccination.
- Participants should be able to comply with blood draws and follow-up and all study requirements.

**Exclusion criteria:**

Participants were excluded from the study if they had:

- History of any meningococcal vaccine administration (naïve participants) or history of any meningococcal vaccine administration other than vaccination given in the parent study (study groups A and B);
- Progressive, unstable or uncontrolled clinical conditions;
- Hypersensitivity, including allergy, to any component of vaccines (including diphtheria toxoid [CRM_197_] and latex) whose use is foreseen in this study;
- Clinical conditions representing a contraindication to intramuscular vaccination and blood draws;
- Abnormal function of the immune system resulting from: clinical conditions; systemic administration of corticosteroids (per oral/intravenous/intramuscular) for more than 14 consecutive days within 90 days prior to enrollment; administration of antineoplastic and immunomodulating agents or radiotherapy within 90 days prior to informed consent; received immunoglobulins or any blood products within 90 days prior to enrollment;
- Received an investigational or non-registered medicinal product within 30 days prior to enrollment;
- Received any other vaccines within 14 days (for inactivated vaccines) or 28 days (for live vaccines) prior to enrollment in this study or who are planning to receive any vaccine within 14 days from the study vaccines;
- Study personnel as an immediate family or household member;
- Who have experienced a moderate or severe acute infection and/or fever (defined as temperature ≥38°C) within 3 days prior to enrollment;
- Who have received systemic antibiotic treatment within 3 days prior to enrollment;
- Any other clinical condition that, in the opinion of the investigator, might interfere with the results of the study or pose additional risk to the individual due to participation in the study.
