# Supplementary material for: Four-year antibody persistence and response to a booster dose of a pentavalent MenABCWY vaccine administered to healthy adolescents and young adults
Source: Hum Vaccin Immunother. 2018 May 9;14(5):1161–74. doi: 10.1080/21645515.2018.1457595 (PMC5989907; doi:10.1080/21645515.2018.1457595)
Supplement: KHVI_A_1457595_Supplemental.zip [file khvi-14-05-1457595-s001.zip › KHVI_A_1457595_Supplemental2.docx]

**Supplementary Material 2**

**Immunogenicity assessment**

All human serum bactericidal assays (hSBA) were performed in GlaxoSmithKline Biologicals, Clinical Laboratory Sciences, Marburg (Germany) and enzyme-linked immunosorbent assays (ELISA) were performed at Public Health England (Manchester, UK) by the Vaccine Evaluation Unit in a blinded manner towards the treatment arm and the visit and participant.

Four blood samples (approximately 20 mL each) were collected from all participants according to the following schedule: day 1 (all groups), day 4 (Groups III, VIa), day 8 (Groups III, VIb), day 31 (all groups), day 34 (subgroup VIIa), day 38 (subgroup VIIb), day 61 (subgroups VIa, VIb, VIIa, VIIb).

Immunogenicity for *N. meningitidis* serogroups A, C, W, Y and serogroup B test strains (H44/76, 5/99, M14459, M10713, M01-0240364, NZ98/254 and 96217) was measured by high throughput serum bactericidal assay using human complement (HT-hSBA), an automated version of the SBA.

Each of the serogroup B test strains measures bactericidal activity directed against one of the major meningococcal bactericidal antigens included in the vaccine: strains H44/76 and M14459 measure serum bactericidal activity against factor H binding protein (fHbp) variant 1.1; strain M07-0241084 measures bactericidal activity against Neisserial Heparin Binding Antigen (NHBA), strains 5/99 and M01-0240364 and 96217 measure bactericidal activity against antigen *Neisseria* adhesin A (NadA); and strain NZ98/254 measures activity against porin A (PorA P1.4), the immunodominant antigen in the outer membrane vesicles (OMV) NZ vaccine component.

For Group III, an overtime assessment of vaccine responses (percentage of participants with hSBA titers ≥5 against serogroup B test strains and hSBA GMTs) starting in the primary study and including each time point from pre-vaccination to post-booster was included in this extension study.
