# Supplementary material for: Four-year antibody persistence and response to a booster dose of a pentavalent MenABCWY vaccine administered to healthy adolescents and young adults
Source: Hum Vaccin Immunother. 2018 May 9;14(5):1161–74. doi: 10.1080/21645515.2018.1457595 (PMC5989907; doi:10.1080/21645515.2018.1457595)
Supplement: KHVI_A_1457595_Supplemental.zip [file khvi-14-05-1457595-s001.zip › KHVI_A_1457595_Supplemental3.docx]

**Supplementary Material 3**

**Determination of sample size**

The sample size for the follow-on groups was determined by the number of participants in the parent study and the previous extension study eligible to participate in this second extension study. The maximum number of participants for each group from the parent study, eligible to participate in this second extension was 48 participants for Group III (who received 2 doses of MenABCWY+OMV in the parent study), and 73 participants for Group VI (who received MenACWY-CRM and placebo in the parent study). Approximately 50 vaccine-naïve participants (similar in age to the follow-on participants) were newly recruited and received 2 doses of MenABCWY+OMV).

From the parent study, the responses (percentages of participants with hSBA≥8) 1 month after receiving one dose of MenABCWY+OMV ranged from 75% to 100% across ACWY serogroups. Assuming a range of observed responses between 75% and 90%, the calculation assumes roughly 85% participant retention in each group. The half-width (precision) of the two-sided 95% confidence intervals (CIs) ranged from: 9% to 13% for Group III; 8% to 11% for Group VI (VIa+VIb); and 9% to 13% for Group VII (VIIa+VIIb).
